# EV1000/VolumeView: a new device for a more reliable measurement of extravascular lung water index in patients with lung resections

**DOI:** 10.1186/cc12130

**Published:** 2013-03-19

**Authors:** A Donati, C Melia, V Monaldi, R Domizi, E Damiani, A Carsetti, C Scorcella, R Castagnani, P Pelaia

**Affiliations:** 1 Università Politecnica delle Marche, Ancona, Italy

## Introduction

Hemodynamic monitoring is important in high-risk surgical patients in order to detect and correct circulatory instability, thereby improving outcome [1]. The extravascular lung water index (EVLWI) reflects pulmonary edema [2]. The new EV1000/VolumeView (Edwards Lifesciences) can accurately measure EVLWI corrected for the actual volume of lung parenchyma (EVLWIc). The aim of our study is to prove a stronger correlation between EVLWIc and PaO_2_/FiO_2 _compared with EVLWI in patients undergoing pulmonary resection.

## Methods

A prospective observational study. Seven patients with lung cancer undergoing pulmonary resection were monitored using the EV1000 plathform. EVLWI was assessed by thermodilution at the following time points: after intubation (t1); during single-lung ventilation (t2); after lung resection (t3); after ICU admission (t4); 12 hours (t5) and 18 hours after ICU admission (t6). EVLWIc values were also collected at t3 and t4. PaO_2_/FiO_2 _was measured at the same time points.

## Results

**Figure 1:**
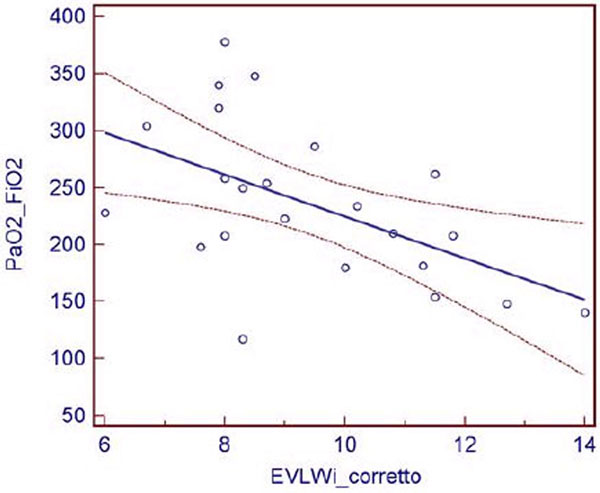
**Correlation between PaO_2_/FiO_2 _and EVLWIc**.

No significant correlation was found between EVLWI and PaO_2_/ FiO_2 _(*r *= -0.3124, *P >*0.05), while a significant correlation was seen between EVLWIc and PaO_2_/FiO_2 _(*r *= -0.528, *P *= 0.009; Figure 1).

## Conclusion

Despite the small sample size, this study shows that in patients undergoing pulmonary resection the EVLWIc is more strongly correlated to PaO2/FiO2 than EVLWI. Therefore, the EV1000 may be a valuable tool for more reliable hemodynamic monitoring in this subgroup of patients.
